# The full blood count as a biomarker of outcome and toxicity in ipilimumab‐treated cutaneous metastatic melanoma

**DOI:** 10.1002/cam4.878

**Published:** 2016-09-29

**Authors:** Leila Khoja, Eshetu G. Atenafu, Arnoud Templeton, Ye Qye, Mary Anne Chappell, Sam Saibil, David Hogg, Marcus O. Butler, Anthony M. Joshua

**Affiliations:** ^1^ Princess Margaret Cancer Centre610 University Avenue. M5G2M9TorontoCanada; ^2^ Astrazeneca plcMelbourn Science ParkDaVinci BuildingMelbournUnited Kingdom; ^3^ Kinghorn Cancer CentreSt Vincents HospitalSydneyAustralia

**Keywords:** Biomarkers, eosinophil to lymphocyte ratio, immune‐related adverse events, ipilimumab, melanoma, neutrophil to lymphocyte ratio, platelet to lymphocyte ratio, toxicity

## Abstract

Ipilimumab produces durable responses in some metastatic melanoma patients. Neutrophil, platelet, and eosinophil to lymphocyte ratios (NLR, PLR, and ELR) may be associated with the immune response in cancer thereby acting as biomarkers of toxicity and efficacy in ipilimumab‐treated patients. Data were collected on clinical characteristics and lactate dehydrogenase (LDH), NLR, PLR, and ELR at baseline, post cycle 2 and at the end of treatment for 183 patients treated with ipilimumab between 2008 and 2015 at the Princess Margaret Cancer Centre. Associations between clinical characteristics, LDH, NLR, PLR, and ELR with toxicity or survival outcomes of progression‐free (PFS) and overall survival (OS) were assessed using univariable and multivariable analysis. Prognostic models of outcome at each time point were determined. Of the 183 patients included, the median age was 58, 85% had M1c disease, 58% were performance status 1, and 64% received ipilimumab as second line therapy. Median follow up was 7.5 months (range: 0.3–49.5), median PFS was 2.8 months (95% confidence intervals (CI): 2.8–3.2), and median OS was 9.6 months (95% CI: 7.9–13.2). Prognostic factors for OS by multivariable analysis were LDH and NLR at all‐time points. Prognostic models using LDH (× 2 upper limit of normal) and NLR 4) differentiated patients into high, moderate, and low risk of death prior to or on ipilimumab treatment (*P* < 0.0001 for each model). No factors were associated with toxicity. Prognostic models based on NLR and LDH values at baseline and on treatment differentiate patients into good, intermediate, and poor prognostic groups and may be relevant in patient management.

## Introduction

Ipilimumab, a CTLA‐4 inhibitor was the first therapy to demonstrate a survival benefit in metastatic melanoma 1. A meta‐analysis of data from 1861 patients from phase II and phase III trials as well as 2985 patients treated on the international expanded access program has confirmed a median overall survival of 9.5 months with long‐term responses beyond 3 years in 21% of patients 2. Ipilimumab is now a standard of care for first and/or second‐line treatment of stage 4 melanoma at the 3 mg/kg dose. More recently, pembrolizumab and nivolumab, anti‐PD‐1 antibodies have shown significantly improved survival in metastatic melanoma as first‐ or higher line of therapy 3, 4, 5. The combination of these two checkpoint inhibitors has additionally shown better responses over either agent alone 6. Nevertheless, personalizing treatment paradigms in metastatic melanoma mandate better prediction of response and outcome with individual agents to maximize their potential benefit and minimize toxicities.

Toxicity seen with checkpoint inhibitors targeting CTLA‐4 or the PD‐1/PD‐L1 axis is immune related, termed immune‐related adverse events (irAE) and can vary in onset and severity 7. Responses, particularly with ipilimumab may be delayed so that progression may occur before a benefit is seen. Such initial progression may be real or pseudo‐progression due to a localized inflammatory reaction, which resolves over time 8. Tumor‐specific mutant antigens have been suggested to predict response to CTLA‐4 blockade 9, 10; however, there are no clinically validated biomarkers of response to ipilimumab. Elevated lactate dehydrogenase (LDH) (double the upper limit of normal (ULN) or 1.5 × ULN) has been proposed as a negative selection criterion for ipilimumab treatment while changes in lymphocyte counts during treatment have been shown to be predictive of response 11, 12, 13, 14. Furthermore, eosinophil counts have been associated with irAE 15. Recently, NLR, in addition to other clinical parameters has shown prognostic importance at baseline prior to ipilimumab treatment, the optimal cut‐off value for prognostication is not defined but has been suggested as 4 or 5 16, 17, 18. Likewise neutrophil, eosinophil, or platelet counts and their ratios to lymphocytes have been shown to be of prognostic value in a wide range of malignancies 19, 20, 21.

Here, we explore the clinical utility of the neutrophil to lymphocyte (NLR), platelet to lymphocyte (PLR), and eosinophil to lymphocyte (ELR) ratios as prognostic biomarkers and their association at baseline and during treatment with outcome and toxicity in patients with metastatic melanoma treated with ipilimumab. Additionally, we aimed to produce prognostic models at specified time points (baseline, post cycle 2, and post completion of treatment) using LDH (cut‐off of × 2ULN) and NLR (cut‐off of 4) based on previous analyses 11, 16, 17, 18.

## Materials and Methods

This retrospective study was conducted at the Princess Margaret Cancer Centre, Toronto, Ontario, Canada using a research ethics board (REB)‐approved protocol (14‐8393‐CE) and was done in accordance with the Declaration of Helsinki. Electronic health records of cutaneous melanoma patients who had received ipilimumab (3 mg/kg or 10 mg/kg) between 2008 and 2015 were reviewed and data collected on sex, age, performance status, tumor burden, previous treatments, mutation status of primary tumors, number of ipilimumab infusions, response on CT scan (evaluated at 4 weeks after the last dose of ipilimumab and once in 3 months thereafter; best response at the time of data cut‐off was used in this study), and survival outcomes of progression‐free survival (PFS) and overall survival (OS). NLR, PLR, and ELR were calculated at the start of ipilimumab (on the day of the first infusion of ipilimumab or within a 2‐week period before treatment), after 6 weeks (i.e., after cycle 2), and at the end of treatment for all patients (4–6 weeks after the last infusion of ipilimumab), whre possible. LDH levels at these time points were also collected.

Chi‐square/Fisher's exact test (as appropriate) were used to assess the contribution of each level to categorical covariates of interest; whereas, logistic regression was used to assess the impact of each of the potential continuous predictors to the binary outcome of toxicity. Time to death and progression were calculated from time of first ipilimumab infusion to date of event (PFS defined as clinical deterioration preventing any further medical treatment, progression on CT scan at defined time point or death). OS and PFS rates were obtained using the Kaplan–Meier method. The log‐rank test was used for univariable analysis to assess the impact of patient characteristics and blood parameters with OS and PFS. Potential covariates that were associated with outcome from univariable‐level (log‐rank test) analysis and other covariates that were clinically relevant were then used to fit the multivariable proportional hazards model to assess joint effect.

Prognostic models for overall survival using NLR and LDH at specific time points during treatment were derived using binary partitioning. Considering the three different (but close cut‐off) points thereby derived (and clinical practice), a summary cut‐off value was considered. The cut‐off values for these parameters were defined as NLR of 4 16, 17, 18 and LDH of 440 U/L 11, in keeping with previous published studies.

Results were considered significant if *P* < 0.05. Statistical analyses were performed using 9.4 of the SAS System for Windows (SAS Institute, Inc., Cary, NC) and the open source statistical software R version 3.0 (The R Foundation for Statistical Computing, Austria, 2013). Full blood count analysis was performed as per standard of procedures at our institution with an automatic cell counter using the coulter principle [normal ranges for full blood count variables were neutrophil 2.0–7.5 × 10^9^, platelet 150–400 × 10^9^, eosinophils 0.04–0.4 × 10^9^, and lymphocytes 1.5–4.0 × 10^9^, LDH range was 125–220 IU]). REMARK guidelines were followed in reporting the results of this study 22.

In order to assess that the prognostic factors were differentiated into different risk sets or not, further analysis in similar such data is crucial to validate findings. In the absence of such data, a bootstrap sample of the original data, resampling of that original data (with replacement), and having the same sample size as the original, can be considered as a test dataset for producing empirical power. Accordingly, a bootstrap sample was drawn from the original dataset 1000 times to calculate the empirical power of the prognostic group developed. Significant level of 0.05 was used to assess the association of the prognostic factor to OS and assigned as yes or no accordingly, for each bootstrap sample. Additional significant levels of 0.01 and 0.001 were also used to summarize the association of the prognostic factor on OS for each bootstrap sample in addition to the usual 0.05 significance level. A summary of the number of times of significant results was calculated. This summary result is the empirical power of the prognostic factor created in differentiating patients into different risk sets.

## Results

### Patient characteristics

A total of 183 patients were identified and had analyzable data for this study. All patients had metastatic cutaneous melanoma. Patients’ characteristics are shown in Table 1. In brief, 63% were male, median age was 58 (range: 24–89 years), 7% had M1a, 8% had M1b, and 85% had M1c disease, 33% were performance status 0, 58% performance status 1 (9% unknown) and ipilimumab treatment was given as first‐line therapy in 5%, second‐line in 65%, third‐line in 23%, and ≥4th line in 7%. Prior treatments to ipilimumab included chemotherapy, high‐dose IL‐2, pembrolizumab, or experimental regimen on clinical trials. The median number of ipilimumab infusions received was 4.

**Table 1:** Patients’ characteristics

Patients’ Characteristics		Number patients*N* = 183 (%)
Age	Median (range)	58 (24–89)
Sex		
Males		115
Females		68
AJCC stage		
M1a		12
M1b		14
M1c		156
Stage III (unresectable)		1
Performance status		
0		61
1		106
2		1
Unknown		15
C‐kit	Mutated/wild type/unknown	0/29/153
BRAF	Mutated/wild type/unknown	61/94/28
NRAS	Mutated/wild type/unknown	26/31/126
Ipilimumab administered		
1st line		10
2nd line		118
3rd line		42
≥4th line		13
Dose of ipilimumab		
3 mg/kg		162
10 mg/kg		21

The median time of follow up was 7.5 months (range: 0.3–49.5 months), whereupon 163 patients (89%) had progressed post ipilimumab treatment and 124 patients (68%) had died at the time of analysis. Median PFS was 2.8 (95% CI: 0.3–35.2) months and median OS was 7.5 (95% CI: 0.3–49.5) months, respectively. Following ipilimumab treatment, 113 (62%) patients did not receive any other therapy; the remaining patients had single or multiple subsequent treatments including pembrolizumab or nivolumab (*n* = 30, 16%), targeted therapy (*n* = 19, 10%), high‐dose IL2 or adoptive cell therapy (*n* = 8, 4%) or were enrolled onto clinical trials (*n* = 14, 8%). Eight (4%) patients received ipilimumab reinduction.

IrAEs were seen in 75 patients (41%) and included rash, hepatitis, deranged thyroid function, diarrhea, and hypophysitis. Steroid treatment was necessary in 49 (27%) patients (for grade 2 or above toxicity) and infliximab treatment in 9 (5%) patients (for grade 3 or above toxicity). Of the 183 patients, 122 (67%) had progressive disease (PD), 28 (15%) had a partial response (PR), 23 (13%) had stable disease (SD), 6 (3%) had a complete response (CR) and 4 (2%) patients did not have an assessment scan on ipilimumab treatment.

Blood parameters of NLR, PLR, ELR, and LDH at the specified time points are shown in Table S1.

### Association of parameters with toxicity and dose

None of the FBC (full blood count) parameters of NLR, PLR, or ELR were significantly associated with toxicity. There were no clinical characteristics associated with increased or decreased toxicity. Additionally, there was no evidence of association between toxicity and response.

A comparison between NLR, PLR, and ELR at all‐time points between patients who received the 3 mg/kg dose (*n* = 162) and those who received the 10 mg/kg (*n* = 21) ipilimumab dose did not reveal enough evidence of differences in these parameters between the doses at any of the time points.

### Associations of response with blood and clinical parameters

LDH at all time points was associated with response; SD, PR, CR (baseline LDH *P* = 0.0003, LDH post cycle 2 *P* = 0.027, and LDH at the end of treatment *P* = 0.001), as was change in LDH post cycle 2 to end of treatment (*P* = 0.023). PLR at all time points was also significant (baseline PLR *P* = 0.023, post C2 PLR *P* = 0.034, and end of treatment PLR *P* = 0.003). NLR at end of treatment was significant for response (*P* = 0.019). Patients with stages M1a and M1b were more likely to respond (66.67%, 57.14%, respectively) than those with M1c disease (26.49%) (*P* = 0.002) as were those with performance status <2 (45.76% for performance status = 0 and 25% for performance status = 1, than 20% for performance status = 2) (*P* = 0.014), Table 2. No other factors including ELR were significant at any time point.

**Table 2:** Factors impacting clinical benefit/response to ipilimumab

Clinical variable	Response		*P*‐value
	CR/PR/SD (*n* = 56)	PD (*n* = 122)	
Continuous variables, median (range)			
Age	60 (24–85)	56 (25–89)	0.53
Baseline LDH, U/L	235 (153–473.00)	277 (93–3345)	**0.003**
LDH post cycle 2	237 (151–826)	269 (128–2086)	**0.027**
LDH at end of treatment	231 (151–415)	308 (130–4366)	**<0.001**
Change in LDH post cycle 2 to end of treatment	−1.00 (−665–179)	49 (−490–3417)	**0.023**
Baseline NLR	3.4 (0.3–19)	3.6 (0.7–37.1)	0.13
NLR post cycle 2	2.4 (0.5–90)	4.2 (0.7–46)	0.40
NLR end of treatment	3.1 (1.0–29.8)	5.6 (0.4–205)	**0.019**
Baseline PLR	182 (96–640)	237 (22–1365)	**0.023**
PLR post cycle 2	140 (55–1380)	222 (39–881)	**0.034**
PLR end of treatment	149 (58–703)	243 (13–790)	**0.003**
Baseline ELR	0.09 (0–0.5)	0.09 (0–1.33)	**0.92**
ELR post cycle 2	0.21 (0–1.27)	0.14 (0–2.6)	**0.56**
ELR end of treatment	0.16 (0–2.77)	0.09 (0–1.0)	**0.14**
Categorical variables, *n* (%)			
Sex (F:M)	23:33	43:79	0.45
Performance status(0:1:2)	27:26:3	32:78:12	**0.014**
AJCC stage (M1a:M1b:M1c:III)	8:8:40	4:6:111:1	**0.002**

CR, complete response; ELR, eosinophil to lymphocyte; LDH, lactate dehydrogenase; NLR, Neutrophil lymphocyte ratios; PD, progressive disease; PLR, platelet lymphocyte ratios; SD, stable disease. Values in bold print are considered statistically significant.

### Prognostic factors by univariable analysis for survival outcomes

Median PFS was 2.8 months (95% CI: 2.8–3.2) and median OS was 9.6 months (95% CI: 7.9–13.2). Factors which were significant by univariable analysis for PFS and OS were performance status, LDH at all‐time points, NLR, and PLR at baseline and at the end of treatment and change in LDH during treatment, Table 3. Change in LDH, NLR, PLR and ELR from baseline to post cycle 2 and from cycle 2 to end of treatment showed that changes in LDH only were prognostic for PFS (*P* < 0.0001 and *P* = 0.02) and OS (*P* < 0.0001 and *P* < 0.001). The prognostic role of LDH remained significant using different cut‐off levels (220 U/L ≤ 440 U/L and finally > 440 U/L).

**Table 3:** Variables impacting prognosis (PFS or OS) by univariable (using log‐rank testing)analysis

Clinical variable	PFS	OS
	Univariate analysis	
Sex	0.14	0.17
Age	0.17	0.26
M stage	0.09	0.08
Performance status	**0.004**	**<0.001**
Baseline LDH	**<0.0001**	**<0.001**
Baseline NLR	**0.02**	**0.003**
Baseline PLR	**<0.0001**	**<0.001**
Baseline ELR	0.46	0.96
LDH after C2	**0.0003**	**<0.001**
NLR after C2	0.65	0.37
PLR after C2	0.21	0.055
ELR after C2	0.18	0.20
LDH at end of treatment	**<0.0001**	**<0.001**
NLR at end of treatment	**<0.0001**	**<0.001**
PLR at end of treatment	**0.0008**	**0.010**
ELR at end of treatment	0.14	0.53
Baseline LDH (220 U/L ≤ 440 U/L and >440 U/L)	0.0003	<.0001
LDH post cycle 2 (220U/L ≤ 440 U/L and >440 U/L)	0.001	<.0001
LDH at the end of treatment (220 U/L ≤ 440 U/L and >440U/L)	<.0001	<.0001

ELR, eosinophil to lymphocyte; LDH, lactate dehydrogenase; NLR, Neutrophil lymphocyte ratios; PLR, platelet lymphocyte ratios. Values in bold print are considered statistically significant.

### A prognostic model for OS to predict outcome at baseline to ipilimumab treatment using NLR and LDH

**Figure 1:**
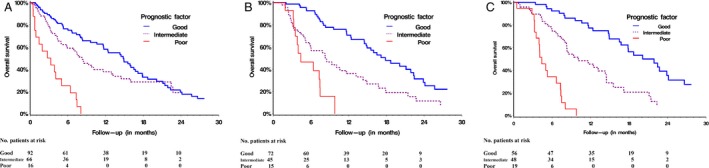
Kaplan–Meier curves of overall survival for each of the prognostic models derived during ipilimumab treatment combining NLR and LDH at each time point. (A) baseline prognostic model (B) prognostic model post cycle 2 of treatment (C) prognostic model at the end of treatment. LDH, lactate dehydrogenase; NLR, Neutrophil lymphocyte ratios.

A baseline model was derived using multivariable analysis followed by binary partitioning. Performance status (HR = 0.41 and 95% CI: 0.21–0.80 for PS = 0, HR = 0.67 and 95% CI: 0.37–1.20 for PS = 1, respectively, with reference to PS = 2, *P* = 0.02), baseline LDH (HR = 1.00 95% CI: 1.00–1.00, *P* < 0.0001), and baseline NLR (HR = 1.04 95% CI: 1.00–1.07, *P* = 0.04) were significant by multivariable analysis. The model discriminated patients into good (LDH < 440 U/L and NLR ≤ 4), intermediate (LDH ≥ 440 U/L and NLR ≤ 4 or LDH < 440 U/L and NLR > 4), and poor (LDH ≥ 440 U/L and NLR > 4) prognostic groups. The 1‐year and 2‐year OS for these groups were 58% (95% CI: 0.46–0.68) and 19% (95% CI: 0.10–0.30) in the good prognostic group, 38% (95% CI: 0.25–0.5) and 19% (95% CI: 0.08–0.34) in the intermediate group, and 0% and 0% in the poor prognostic group, respectively, Figure 1A, Table 4.

**Table 4:** Prognostic patient groups for OS (CI: confidence intervals, survival is shown in months, values are given to the nearest decimal point)

Prognostic Model and groupings	Median OS	95% CI of median	*P* ‐value (log‐rank)	1 year OS (%)	95% CI of 1 year OS	2 year OS (%)	95% CI of 2 year OS
Baseline model							
Good (reference)	14.8	11.60–16.76	<0.0001	58	0.46–0.68	19	0.10–0.30
Intermediate	8.3	4.93–11.63		38	0.25–0.50	19	0.08–0.34
Poor	3	0.72–4.99		0		0	
Post cycle 2 model							
Good (reference)	17.8	14.49–22.34	<0.0001	71	0.57–0.80	28	0.15–0.42
Intermediate	8.3	5.19–13.17		36	0.22–0.50	11	0.03–0.26
Poor	4.4	3.25–7.43		0		0	
End of treatment model							
Good (reference)	20.1	16.49–24.15	<0.0001	77	0.63–0.87	35	0.20–0.51
Intermediate	10.1	7.89–14.46		45	0.30–0.60	8	0.02–0.23
Poor	4.4	3.25–6.67		0		0	

### A prognostic model for OS to predict outcome after cycle 2 ipilimumab using NLR and LDH

Multivariable analysis was then conducted to include parameters post cycle 2; performance status (HR = 0.38 95% CI: 0.165–0.871, for PS = 0 and HR = 0.64 95% CI: 0.30–1.36 for PS = 1, respectively, with reference to PS = 2, *P* = 0.04), baseline LDH (HR = 1.00 95% CI: 0.999–1.00, *P* = 0.86), LDH post cycle 2 (HR = 1.00 95% CI: 1.00–1.00, *P* = 0.007), baseline NLR (HR = 0.95 95% CI: 0.87–1.05, *P* = 0.30), and post cycle 2 NLR (HR = 1.12 95% CI: 1.07–1.17, *P* < 0.0001). A second model after cycle 2 discriminated patients into good (LDH < 440 and NLR ≤ 4), intermediate (LDH ≥ 440 and NLR ≤ 4 or LDH < 440 and NLR > 4), and poor (LDH ≥ 440 and NLR > 4) prognostic groups. The 1‐year and 2‐year OS values for these groups were 71% (95% CI: 0.22–0.50) and 28% (95% CI: 0.15–0.42) in the good prognostic group, 36% (95% CI: 0.22–0.50) and 11% (95% CI: 0.03–0.26) in the moderate group, and 0% and 0% in the poor prognostic group, respectively, Figure 1B, Table 4.

### A prognostic model for OS to predict outcome at the end of ipilimumab treatment using NLR and LDH

Finally, further multivariable analysis using parameters at the end of treatment showed significant prognostic factors to be baseline LDH (HR = 1.00 95% CI: 1.00–1.00, *P* = 0.007), LDH at end of treatment (HR = 1.00 95% CI: 1.00–1.00, *P* < 0.001), baseline NLR (HR = 1.06 95% CI: 1.01–1.10 *P* = 0.016), and NLR at end of treatment (HR = 1.06 95% CI: 1.02–1.09, *P* < 0.001). A third prognostic model at the end of treatment discriminated patients into good (LDH < 440 and NLR ≤ 4), moderate (LDH ≥ 440 and NLR ≤ 4 or LDH < 440 and NLR > 4), and poor (LDH ≥ 440 and NLR > 4) OS groups. The 1‐ and 2‐year OS values for these groupings were 77% (95% CI: 0.63–0.87) and 35% (95% CI: 0.20–0.51) in the good prognostic group, 45% (95% CI: 0.30–0.60) and 8% (95% CI: 0.02–0.23) in the intermediate group, and 0% and 0% in the poor prognostic group, respectively, Figure 1C, Table 4.

All models were validated using bootstrapping (Table S1–S3). The multivariable analysis at each time point is shown in Table S3A and B.

## Discussion

The approval of targeted agents and checkpoint inhibitors for the treatment of metastatic melanoma has significantly improved patient outcomes. Treatment paradigms are evolving; and hence optimal sequencing or combinations of agents may be key to further improving of survival.

The full or complete blood count may be a marker of inflammation and the adaptive immune response in carcinoma. Tumor infiltrating lymphocytes are associated with a good prognosis in a number of tumor types 23 and inflammation may be immune stimulating with stimulatory cytokines such as IFN*γ* or immune suppressive with macrophage, neutrophil infiltration, and production of IL‐8 among other cytokines 24. NLR, PLR, and ELR may serve as surrogate markers of this response prior to and during treatment. Several studies have suggested one or more of these parameters in conjunction with other markers, such as CD4 + , CD8 +  T cells, number of Treg cells, and number of myeloid‐derived suppressor cells (MDSC) as predictive for outcome with ipilimumab 25, 26. A rise in absolute lymphocyte count may well predict for benefit from ipilimumab 14 but may also fail to account for immune suppressive versus stimulatory interaction. Several studies in numerous carcinomas have determined a prognostic role for NLR and PLR but a pharmacodynamic and predictive role on treatment has not been defined 20, 21. It is likely that a panel of markers will be needed to appreciate the complexity of immune‐tumor interactions and multiparameter analysis is needed to determine these factors 27, 28.

Our study is the largest study to examine NLR, PLR, and ELR ratios as potential biomarkers of clinical value at baseline and during treatment with ipilimumab for metastatic melanoma. The prognostic scores derived differentiated patients into poor, intermediate, and good prognostic groups at baseline, during and at the end of ipilimumab treatment. OS is a valid endpoint given the kinetics of response to ipilimumab; especially, in our dataset where 70% of patients had no further treatment. Our prognostic scores could serve to select patients for ipilimumab treatment or as a surrogate pharmacodynamic marker of the immune system (based on NLR) and tumor response during ipilimumab treatment (LDH).

The number of active agents in metastatic melanoma is increasing and hence predictive biomarkers will be crucial to determine treatment paradigms. While combination of agents is an attractive strategy, toxicity can be significant making such treatment intolerable in some patients. Sequential therapy may limit toxicity but could be detrimental to outcome if disease progresses rapidly prohibiting later therapy with more efficacious agents 29. This is particularly relevant to ipilimumab treatment where the response may be delayed. Potential combinations include targeted agents, different checkpoint inhibitors or treatment modalities such as radiotherapy. BRAF inhibition increases tumor infiltrating lymphocytes, putative markers of T‐cell exhaustion, and PD‐ L‐1 expression 30 and different BRAF inhibitors may produce differential effects on lymphocyte counts 31, the implications of which are unclear regarding the use of these ratios in sequencing or combining treatments.

Our study is limited by its retrospective nature and our patient cohort was homogenous (only cutaneous melanomas were examined) but heterogeneous as to what line of therapy ipilimumab was given at and the dose of ipilimumab (14% of patients had the 10 mg/kg dose). Survival outcomes, however, were similar in published trials evaluating ipilimumab as second‐line or higher therapy compared to first line; there were no differences in outcomes between the two doses 1, 32.

In summary, we have further validated LDH and NLR as independent prognostic biomarkers in metastatic melanoma. Our prognostic scores may be clinically useful but will require independent validation. We did not find any associations of ELR or PLR with toxicity or response. Neither parameter was prognostic in multivariable analysis.

## Conflicts of Interest

Arnoud Templeton has served as a consultant (no personal compensation) with BMS, D. Hogg serves on Advisory Boards for Roche, Merck, GlaxoSmithKline, Bristol‐Myers Squibb, and Novartis. Dr. Butler has served on advisory boards for Merck and has speaker honaria from Merk Canada and BMS Canada, Leila Khoja is an employee of AstraZeneca plc but this work is independent of AstraZeneca.

## Supporting information


**Table S1.** Baseline, post cycle 2, and end of treatment levels of LDH, NLR, PLR, and ELRs (median, range, and interquartile ranges—IQR are shown, the total number of patients was 183).
**Table S2.** Summary results based on 1000 bootstrap samples. (A) Significant results by prognostic score (using level of 0.05). (B) Significant results by prognostic score (using level of 0.01). (C) Significant results by prognostic score (using level of 0.001). (D) Summary of P‐values (ProbChiSq Pr > Chi‐Square).
**Table S3.** Multivariable analysis at each time point in the study. (A) Variables impacting on prognosis (PFS) by multivariable analysis.(B) Variables impacting on prognosis (OS) by multivariable analysis.
