# Supplementary material for: The full blood count as a biomarker of outcome and toxicity in ipilimumab‐treated cutaneous metastatic melanoma
Source: Cancer Med. 2016 Sep 29;5(10):2792–9. doi: 10.1002/cam4.878 (PMC5083732; doi:10.1002/cam4.878)
Supplement: Supplementary file 1 — Table S1. Baseline, post cycle 2, and end of treatment levels of LDH, NLR, PLR, and ELRs (median, range, and interquartile ranges—IQR are shown, the total number of patients was 183). Table S2. Summary results based on 1000 bootstrap samples. (A) Significant results by prognostic score (using level of 0.05). (B) Significant results by prognostic score (using level of 0.01). (C) Significant results by prognostic score (using level of 0.001). (D) Summary of P‐values (ProbChiSq Pr > Chi‐Square). Table S3. Multivariable analysis at each time point in the study. (A) Variables impacting on prognosis (PFS) by multivariable analysis.(B) Variables impacting on prognosis (OS) by multivariable analysis. [file CAM4-5-2792-s001.docx]

Supplementary Table 1. Baseline, post cycle 2 and end of treatment levels of LDH, NLR, PLR and ELRs (median, range and interquartile ranges IQR are shown, the total number of patients was 183)

| **Biomarker** | | **Baseline** | **Post cycle 2** | **End of treatment** |
| --- | --- | --- | --- | --- |
| LDH | Median | 254 | 250 | 281 |
|  | Range | 93-3345 | 128-2086 | 130-4366 |
|  | IQR | 202-348 | 202.5-339.5 | 220-386 |
|  | Number of missing values | 5 | 51 | 46 |
| NLR | Median | 3.50 | 3.64 | 4.33 |
|  | Range | 0.30-37.14 | 0.52-90.0 | 0.42-205 |
|  | IQR | 2.37-5.59 | 2.24-5.79 | 2.71-7.29 |
|  | Number of missing values | 6 | 30 | 53 |
| PLR | Median | 215 | 185.45 | 187.90 |
|  | Range | 22.31-1365 | 39.17-1380 | 12.81-790 |
|  | IQR | 140.09-314.62 | 116.15-288.28 | 125.41-281.43 |
|  | Number of missing values | 7 | 30 | 54 |
| ELR | Median | 0.09 | 0.17 | 0.12 |
|  | Range | 0.00-1.33 | 0.00-2.60 | 0.00-2.77 |
|  | IQR | 0.02-0.17 | 0.08-0.29 | 0.04-0.23 |
|  | Number of missing values | 6 | 30 | 54 |

Summary results based on 1000 bootstrap samples

Table 2a significant results by prognostic score (using level of 0.05)

|  | Prognostic | | |
| --- | --- | --- | --- |
|  | Baseline | Post cycle 2 | End of treatment |
| Significant | 999 | 1000 | 1000 |

Table 2b significant results by prognostic score (using level of 0.01)

|  | Prognostic | | |
| --- | --- | --- | --- |
|  | Baseline | Post cycle 2 | End of treatment |
| Significant | 997 | 1000 | 999 |

Table 2c significant results by prognostic score (using level of 0.001)

|  | Prognostic | | |
| --- | --- | --- | --- |
|  | Baseline | Post cycle 2 | End of treatment |
| Significant | 993 | 1000 | 998 |

Table 2d Summary of P-values (ProbChiSq Pr > Chi-Square)

| Prognostic score | N | Median | Minimum | Maximum |
| --- | --- | --- | --- | --- |
| Baseline | 1000 | 3.1481329E-9 | 1.902946E-23 | 0.0821892 |
| Post cycle 2 | 1000 | 9.895904E-17 | 6.06203E-35 | 8.25375E-7 |
| End of treatment | 1000 | 3.18582E-10 | 6.402492E-24 | 0.0120335 |

Supplementary Table 3 Multivariable analysis at each time point in the study.

***Supplementary Table 3A. Variables impacting on prognosis (PFS) by multivariable analysis***

| **Variable** | **Level** | **At Baseline** | | **After C2** | | **At end of treatment** | |
| --- | --- | --- | --- | --- | --- | --- | --- |
|  |  | **P-value** | **HR**  **(95% CI)** | **P-value** | **HR**  **(95% CI)** | **P-value** | **HR**  **(95% CI)** |
| PS (Ref=2) | 0 | 0.02 | 0.43 (0.23-0.81) | 0.12 | 0.62 (0.3-1.30) | 0.056 | 0.44 (0.20-0.94) |
|  | 1 |  | 0.62 (0.34-1.1) |  | 0.95 (0.47-1.93) |  | 0.67 (0.33-1.35) |
| LDH (ref=>440) | <220 | 0.01 | 0.46 (0.28-0.77) | 0.03 | 0.47 (0.27-0.83) | <0.0001 | 0.18 (0.09-0.35) |
|  | > 220 and  < 440 |  | 0.55 (0.34-0.88) |  | 0.60 (0.36-1.02) |  | 0.35 (0.20-0.61) |
| NLR |  | 0.93 | 1.00 (0.96-1.05) | 0.004 | 1.08 (1.02-1.13) | 0.015 | 0.95 (0.91-0.99) |
| ELR |  | 0.56 | 0.66 (0.19-2.31) | 0.50 | 0.78 (0.38-1.60) | 0.03 | 0.27 (0.08-0.87) |
| PLR |  | 0.01 | 1.00 (1.00-1.003) | 0.69 | 1.00 (0.1-1.00) | 0.002 | 1.003 (1.00-1.01) |

***Supplementary Table 3B. Variables impacting on prognosis (OS) by multivariable analysis***

| **Variable** | **Level** | **At Baseline** | | **After C2** | | **At end of treatment** | |
| --- | --- | --- | --- | --- | --- | --- | --- |
|  |  | **P-value** | **HR**  **(95% CI)** | **P-value** | **HR**  **(95% CI)** | **P-value** | **HR**  **(95% CI)** |
| PS (Ref=2) | 0 | 0.02 | 0.43  (0.22-0.85) | 0.005 | 0.27 (0.12-0.61) | 0.053 | 0.4 (0.18-0.88) |
|  | 1 |  | 0.76  (0.42-1.39) |  | 0.49 (0.24-1.01) |  | 0.66 (0.33-1.34) |
| LDH (ref=>440) | <220 | <.0001 | 0.25  (0.14-0.45) | 0.008 | 0.39 (0.21-0.73) | <0.0001 | 0.12 (0.05-0.27) |
|  | > 220 and  < 440 |  | 0.35  (0.21-0.6) |  | 0.44 (0.24-0.79) |  | 0.29 (0.15-0.53) |
| NLR |  | 0.72 | 0.99  (0.94-1.04) | 0.03 | 1.06 (1.01-1.11) | 0.011 | 1.05 (1.01-1.10) |
| ELR |  | 0.69 | 0.73  (0.16-3.44) | 0.12 | 0.61 (0.32-1.14) | 0.65 | 0.73 (0.18-2.89) |
| PLR |  | 0.10 | 1.001  (1.00-1.003) | 0.05 | 1.00 (1.000-1.005) | 0.53 | 0.10 (0.10-1.002) |
